# Survey of commercial *Rhodiola* products revealed species diversity and potential safety issues

**DOI:** 10.1038/srep08337

**Published:** 2015-02-09

**Authors:** Tianyi Xin, Xiaojin Li, Hui Yao, Yulin Lin, Xiaochong Ma, Ruiyang Cheng, Jingyuan Song, Lianghong Ni, Congzhao Fan, Shilin Chen

**Affiliations:** 1 Institute of Medicinal Plant Development, Chinese Academy of Medical Sciences & Peking Union Medical College, Beijing 100193, P.R. China; 2 Xinjiang Institute of Chinese Materia Medica and Ethnical Materia, Urumqi 830002, P.R. China; 3 Institute of Chinese Materia Medica, China Academy of Chinese Medical Sciences, Beijing 100700, P.R. China; 4 Chongqing Institute of Medicinal Plant Cultivation, Chongqing 408435, P.R. China; 5 Shanghai University of Traditional Chinese Medicine, Shanghai 201203, P.R. China

## Abstract

The adulteration of herbal products is a threat to consumer safety. Here we surveyed the species composition of commercial *Rhodiola* products using DNA barcoding as a supervisory method. A *Rhodiola* dietary supplement DNA barcode database was successfully constructed using 82 voucher samples from 10 *Rhodiola* species. Based on the DNA barcoding standard operating procedure (SOP), we used this database to identify 100 Rhodiolae Crenulatae Radix et Rhizoma decoction piece samples that were purchased from drug stores and hospitals. The results showed that only 36 decoction piece sequences (40%) were authentic *R. crenulata*, which is recorded in Chinese Pharmacopeia, whereas the other samples were all adulterants and may indicate a potential safety issue. Among the adulterants, 35 sequences (38.9%) were authenticated as *R. serrata*, nine sequences (10%) were authenticated as *R. rosea*, which is documented in the United States Pharmacopeia, and the remaining samples were authenticated as other three *Rhodiola* species. This result indicates decoction pieces that are available in the market have complex origins and DNA barcoding is a convenient tool for market supervision.

In recent years, the general public and media have paid significant attention to the efficacy of dietary supplements in preventing chronic diseases and improving health. Herbal medicines and other natural remedies have also received additional attention and are more popular than ever. At the International Conference on Traditional Medicine for South-East Asian Countries in February 2013, the World Health Organization (WHO) Director General, Dr. Margaret Chan, stated that “traditional medicines of proven quality, safety, and efficacy, contribute to the goal of ensuring that all people have access to care”^1^. According to the WHO, the adulteration of herbal products is a threat to consumer safety^2^. Under the Dietary Supplement Health and Education Act (DSHEA) of 1994, manufacturers and distributors of dietary supplements are prohibited from marketing products that are adulterated or misbranded, and these firms are responsible for evaluating the safety and labeling of their products before marketing to ensure that they meet all of the requirements of the DSHEA and all regulations of the United States Food and Drug Administration (FDA)^3^. Currently, steps have been taken to strengthen the provisions in the regulatory framework for dietary supplements, especially in relation to Good Manufacturing Practices and adverse event reporting in the United States.

As a dietary supplement, the phytochemical extracts of *Rhodiola* species are widely used throughout Europe, Asia, and the United States, with biological activities that include anti-allergenic and anti-inflammatory effects, enhanced mental alertness, and a variety of other therapeutic applications^4^. In the consolidated list of Article 13 health claims of the European Food Safety Authority (EFSA), *Rhodiola* dietary supplements are listed as “good for handling of physical and mental stress”^5^.

One of the most widely used *Rhodiola* species in American and European countries is *R. rosea*, and it has a long history as a valuable medicinal plant. In the United States Pharmacopeia, *R. rosea* is recorded as Rhodiola rosea Root and Rhizome, and potentially confounding materials include *R. kirilowii*, *R. yunnanensis*, *R. crenulata*, *R. sacra*, and *R. sachalinensis*^6^. According to Galambosi, over 46 companies worldwide sell *R. rosea* products, and there are 30 companies that supply these products as food ingredients^7^. The rapidly growing demand for raw material has caused *R. rosea* to be a threatened plant in many countries^8^,^9^,^10^,^11^; therefore, substitutions have appeared. There are approximately 24 different species of the genus *Rhodiola* growing in the Altay region that can be misclassified as *R. rosea*^12^.

*R. crenulata*, in contrast, is the authentic Rhodiolae Crenulatae Radix et Rhizoma species that is accepted by Chinese Pharmacopoeia^13^. *R. crenulata* is a relatively rare perennial herbaceous plant that is primarily distributed in several of the harshest environments in the world, including eastern Tibet, northern Yunnan, and western Sichuan. Its dried root and rhizome are called “Hongjingtian” in Chinese. In Asia, it is called "Plateau Ginseng," and it is considered to be a sacred herb by the Tibetans. This incredible herb has been used to enhance inner spiritual power, concentration and physical endurance. Since the 1980s, the accelerated and uncontrolled use of *R. crenulata* in southwestern China has led to deforestation, with a number of *Rhodiola* species, including *R. crenulata*, being considered for inclusion in the National Class One Endangered Species in China checklist for conservation purposes^14^. Because of the rapidly increasing commercial utility of the raw materials, decoction pieces, and dietary supplements of this species, other species of *Rhodiola* have been sold as Rhodiolae Crenulatae Radix et Rhizoma in the market.

The genus *Rhodiola* L. (Crassulaceae) is found in the alpine regions of Asia and Europe^15^, and their medicinal material morphology and tissue structure are similar; thus, distinguishing these species merely by sight is impossible for those who are untrained (Figure 1). Therefore, various techniques, such as microscopy^16^, chemotaxonomic classification by high-performance liquid chromatography-diode array detector/ultraviolet (HPLC-DAD/UV)^17^, high-performance liquid chromatography-post-acceleration detector-electron impact-mass spectrometry (HPLC-PAD-EI-MS)^18^, and inter simple sequence repeat (ISSR)^14^, have emerged to distinguish *R. crenulata* from its closely related species. Different molecular markers have also been used to study the genetic variation in *Rhodiola* species. Guest surveyed the variation in a nuclear (internal transcribed spacer - ITS) and chloroplast (*psbA-trnH* spacer) DNA region in *R. integrifolia*, *R. rosea*, and *R. rhodantha*^19^. Mayuzumi suggested that the *trnL-trnF* intergenic spacer and ITS region could be used to infer the phylogenetic position of Eastern Asian Sedoideae^20^. The plastid DNA intergenic spacer *rpl20-rps12* and *trnS-trnG* and nuclear ribosomal internal transcribed spacer (ITS) region were used to explore the genetic variation and phylogeographical history of *R. alsia*^21^. Chloroplast DNA sequences that include *psbA-trnH*, *matK*, *trnC-trnD*, and *trnL-trnF* have been utilized to investigate the phylogeographical patterns of *R. dumulosa*^22^. There are limited reports that involve *R. crenulata* and the other raw materials. Alternatively, DNA barcoding is a useful and powerful tool for nonprofessional users, such as customs officers, traditional drug producers and managers, and forensic specialists^23^. The Chinese Plant BOL Group suggested that the ITS region can serve as a core barcode^24^, and the ITS2 region has been proposed as a standard DNA barcode for medicinal plant authentication^25^,^26^,^27^. Therefore, we aimed to use the ITS2 region as the core barcode to determine the species composition of the *Rhodiola* dietary supplements that are currently available in the market. This study indicated that DNA barcoding is a convenient tool for market supervision.

## Results

### Amplification and sequencing success

Genomic DNA was extracted from the 32 authenticated specimens and 50 Rhodiolae Crenulatae Radix et Rhizoma samples (Table S1). Twenty-two percent of the medicinal material samples contained DNA concentrations greater than 100 ng/μL, 64% contained between 20 and 100 ng/μL, and only 14% were lower than 10 ng/μL (Table S1). Although a small percentage of the samples had a poor DNA yield (lower than 5 ng/μL), the PCR amplification was not affected and all of the medicinal material samples were able to produce PCR products that satisfied the sequencing requirements. The PCR products were successfully sequenced, and high-quality bidirectional sequences were obtained.

Among the 100 test samples, PCR products were successfully obtained from 90% of the samples. An electrophoretic analysis revealed that the genomic DNA fragments of the samples that failed in the PCR amplification were primarily distributed below 250 bp. This result indicates that the degree of genomic DNA degradation in the decoction pieces was higher than in the medicinal materials.

### Establishment of the *Rhodiola* dietary supplement species DNA barcode database

To establish the barcode database, 32 ITS2 sequences were obtained from 10 *Rhodiola* species. The characteristics of these species are summarized in Table 1. The results suggest that the ITS2 sequence length of the 10 species was 227 bp after alignment, and there were 35 variable sites (Figure S1). The average interspecific distance between the 10 species was 0.044 (0-0.109). All 32 of the sequences served as standard sequences in the *Rhodiola* dietary supplement species DNA barcode database.

The 50 authenticated raw material samples were then used to verify the identification stability and accuracy of the standard DNA barcode database. The ITS2 sequence characteristics are also shown in Table 1. There are two variable sites in *R. crenulata* that were used to divide the 50 sequences into three haplotypes (Table S1). The results showed that according to the DNA barcoding SOP, the use of the ITS2 sequence as a DNA barcode can clearly distinguish these species; therefore, all 50 of the medicinal material sequences can also be incorporated into the *Rhodiola* dietary supplement species DNA barcode database.

### Identification of the commercial samples

A total of 100 test samples were purchased from drug stores (89%) and hospitals (11%). All of the samples were labeled as “Hongjingtian” and available to patients and consumers. All 100 samples were used in the DNA resolution, and 90% of the samples contained the ideal genomic DNA for the sequence amplification. Using PCR and bidirectional sequencing, a total of 90 ITS2 sequences were obtained. The length of all 90 sequences was 216–219 bp before the alignment. There were 21 variable sites after the alignment, and the degree of sequence variation among all of the decoction pieces was greater than the intraspecific divergence of *R. crenulata*. This result indicates that the commercial Rhodiolae Crenulatae Radix et Rhizoma decoction pieces are not derived from a single species.

**Figure 1:**
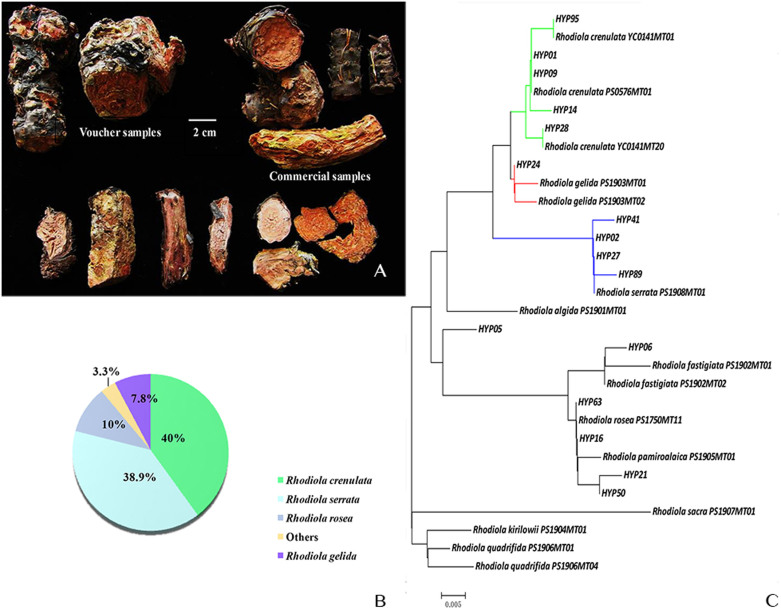
DNA barcoding identification of commercial *Rhodiola* products. (A) Voucher and commercial samples of Rhodiolae Crenulatae Radix et Rhizoma. (B) Species proportions of the commercially available decoction pieces in the market. (C) NJ tree constructed using the ITS2 sequence haplotypes of the voucher and commercial decoction piece samples.

Using the established database, we confirmed the best-hit species of the above-mentioned 90 test samples based on the DNA barcoding SOP. The results showed that 36 (40%) were authenticated as *R. crenulata*, 35 (38.9%) were authenticated as *R. serrata*, and nine (10%) were authenticated as *R. rosea* (Figure 1). In addition, there were seven test sample sequences that showed a high similarity (99%) to *R. gelida* and *R. crenulata*, and the remaining samples matched other *Rhodiola* species (Table S2). The sequence alignment results showed that seven of the above-mentioned samples contained the same haplotype (marked as HYP24), and each contained one variable site compared with the *R. crenulata* haplotype RC2 (site 131 C/T transition) and *R. gelida* haplotype RG2 (site 63 T/C transition). Based on the neighbor-joining (NJ) tree (Figure 1) that was constructed using the ITS2 sequence haplotypes of the voucher samples and the tested decoction piece samples, we found that the decoction piece haplotype HYP24 and *R. gelida* haplotypes clustered into one clade. This study indicates that the most common species found in the commercial Rhodiolae Crenulatae Radix et Rhizoma decoction pieces that are available in the market are *R. crenulata* and *R. serrata*.

## Discussion

### The reliability of the *Rhodiola* dietary supplement species DNA barcode database

This study was the first to use DNA barcoding to detect the species composition of *Rhodiola* dietary supplements that are currently available in the market. Compared with previous reports^19^,^20^,^21^,^22^, we acquired the genomic DNA by using medicinal parts and decoction piece samples instead of fresh or gel-dried leaves. Our experiment demonstrated that genomic DNA can be isolated from the dried roots, and its quality was satisfactory for the subsequent PCR reactions.

We used 82 voucher samples to verify the reliability of the *Rhodiola* dietary supplement species DNA barcode database. All of the samples were authenticated by several authoritative experts from different institutes. All of the ITS2 sequences were confirmed to be reliable and thus were incorporated into the database to analyze the intraspecific and interspecific divergence. The results demonstrated that the ITS2 region of the ten *Rhodiola* species possessed a higher average interspecific divergence than the average intraspecific divergence within each species. Our results highlighted the universality of using the ITS2 sequence as a DNA barcode. The NJ tree (Figure S2) of the 82 voucher samples that was constructed using the ITS2 sequence showed that each species clustered into its own clade and was clearly separated from the other species, which demonstrates that the ITS2 barcode was able to accurately and swiftly distinguish the ten species. This result indicates that the database is highly reliable. For these reasons, we consider the ITS2 barcode to be the most suitable marker for the identification of the *Rhodiola* species used in this study. The *Rhodiola* dietary supplement species DNA barcode database constructed in this study is able to successfully identify the raw materials of the commercial *Rhodiola* dietary supplements and decoction pieces that are currently available in the market.

### Species composition of the raw materials in *Rhodiola* dietary supplements and decoction pieces available in the market

Among the 100 herbal pieces sold in drug stores and hospitals in China as Rhodiolae Crenulatae Radix et Rhizoma, we utilized the above-mentioned database and confirmed that only 40% of the samples were authentic *R. crenulata*, whereas the other samples were all adulterants according to Chinese Pharmacopeia. A total of 38.9% of the test samples were authenticated as *R. serrata*, and the remaining samples matched other *Rhodiola* species. This result indicates that the Rhodiolae Crenulatae Radix et Rhizoma decoction pieces that are available in the market possess complex origins, and the samples purchased from drug stores and hospitals contain adulterants. Among the adulterants, nine samples (10%) were authenticated as *R. rosea,* which is recorded in the United States Pharmacopeia. In addition, 10% of the test samples failed to produce ITS2 sequences. After careful verification, we found that some of the samples were moth-eaten and discolored, which indicates that these samples were improperly stored, thus altering the quality of the decoction pieces. In general, because of the harvest, storage, and transport processes, including the drying temperature, mode of processing, and storage environment and time, the genomic DNA may become seriously degraded and incapable of successful amplification. This result indicates that the quality of the raw material of the dietary supplement did not conform to the requirements.

The detection of Rhodiolae Crenulatae Radix et Rhizoma raw materials and decoction pieces sold in the market also indicated a potential safety issue. It has been reported that cyanogenic glycoside compounds, such as lotaustralin, epilotaustralin, and rhobupcyanoside A, have been isolated from certain *Rhodiola* species (Table S3)^28^,^29^,^30^,^31^. These types of compounds may degrade when in contact with β-glucosidase and α-hydroxynitrile lyase and release hydrogen cyanide (HCN). This study found that in addition to *R. crenulata*, the commercial decoction pieces also included *R. serrata*, *R. rosea*, *R. gelida*, and *R. quadrifida*; the latter three species have all been reported to contain lotaustralin. The use of these species as dietary supplements may be harmful to public health and detrimental to patients and consumers with legitimate interests. Our study indicated that the DNA barcoding technology was able to quickly differentiate these species and effectively identify Rhodiolae Crenulatae Radix et Rhizoma from its adulterants, thereby guaranteeing clinical drug safety and ensuring the vital interests of patients. Therefore, the China Food and Drug Administration must monitor commercial *Rhodiola* dietary supplements. This DNA barcoding technology represents a direct application for supervisory institutions and ensures the legitimate rights and interests of the consumers.

### Future applications of DNA barcoding for market supervisors

DNA barcoding has been used in several applications, such as for tea, fish, seafood, herbal products, and commercialized medicinal plants and animals^32^,^33^,^34^,^35^,^36^,^37^. Researchers believe that DNA barcoding represents a renaissance for the identification of herbal medicines^27^. In addition to the database constructed in this study, another preliminary system for DNA barcoding of herbal materials has already been established based on a two locus combination of the ITS2 + *psbA*-*trnH* barcodes^27^. There are 78,847 sequences belonging to 23,262 species in the system, and they include more than 95% of the crude herbal drugs in published pharmacopeias, including those from China, Japan, Korea, India, the United States, and Europe. All of the sequences determined in this study were entered into this system as well. These sequences provide an effective means for the supervisory institutions to monitor dietary supplements and the herbal medicine market.

Here, we demonstrated how to use the database in a market supervisory setting. The result indicated that the above-mentioned 90 test samples could be confirmed as best-hit species based on the DNA barcoding SOP. For the HYP24 haplotype, which contains one variable site compared with the *R. crenulata* haplotype RC2 and *R. gelida* haplotype RG2, we were able to determine the species of the decoction pieces using an NJ tree (Figure 1) because they clustered into one clade with the *R. gelida* haplotypes and showed a closer genetic relationship with *R. gelida* than *R. crenulata*. This application showed that, based on the current database and in accordance with DNA barcoding SOP, we can successfully implement this authentication method for use with commercial samples.

Because several *Rhodiola* species are threatened or endangered in many countries, this study broadens the application of the ITS2 region to endangered plants. The medicinal plant trade is the primary source of income for herbalists, and economic constraints may provide incentives for herbalists to substitute cheaper and more readily available species for rare ingredients and sell them under the same name^34^. The current situation of domestic food and drug safety is serious, and important issues related to food and drug safety have changed the focus from “illegal addition” to “process control” and “material safety”. The quality control of raw materials may be the key to solving this problem, which has been verified through a consensus among all sectors of the community. DNA barcoding may help realize this authentication goal by monitoring species of raw materials throughout the industrial pipeline. It is an effective strategy for certifying the origin of raw materials and detecting adulterants in the herbal medicine market. Therefore, it is useful for both producers and consumers because the former are interested in certifying their raw materials, whereas the latter are interested in the safety of their medicines. In this study, we successfully utilized this technology to survey a *Rhodiola* dietary supplement sold in the market, and the database could be used for the supervision of other species and for similar work by the supervisory institutions in the future. DNA barcoding will play an important role in the import and export customs inspection process and in drug safety monitoring. In addition, the use of DNA barcoding for market supervision can broaden the application of this technology and provide an excellent inspection method for the supervisory institutions. In conclusion, this study substantially contributes to the protection of public health worldwide.

## Methods

### Sampling of the plant materials

In this study, 82 authenticated samples were used to establish a standard DNA barcode database. These samples include 32 *Rhodiola* specimen samples collected from different habitats (10 species: *R. crenulata*, *R. rosea*, *R. algida* (Ledeb.) Fisch. et Mey., *R. fastigiata* (Hook. f. et Thoms.) S. H. Fu, *R. gelida* Schrenk, *R. kirilowii* (Regel) Maxim., *R. pamiro-alaica* A. Bor., *R. quadrifida* (Pall.) Fisch. et Mey., *R. sacra* (Prain ex Hamet) S. H. Fu, and *R. serrata* H. Ohba) and 50 authenticated Rhodiolae Crenulatae Radix et Rhizoma samples that were gathered from Sichuan and Tibet (Table S1). All of the corresponding voucher samples were deposited in the Herbarium of the Institute of Medicinal Plant Development, Chinese Academy of Medical Sciences, Beijing, China. All of the ITS2 sequence haplotypes of each species were submitted to GenBank under the following accession numbers: KJ796854-KJ796867 (Table S1).

In addition to the voucher samples, we attempted to collect at least three Rhodiolae Crenulatae Radix et Rhizoma decoction piece samples from each province in China, and 100 samples were gathered from drug stores and hospitals in 31 different provinces (Table S2). All of the test samples are readily available to consumers and patients. Using the barcode database, we tested the species composition of the commercial Rhodiolae Crenulatae Radix et Rhizoma that is available in the market.

### Genomic DNA extraction

The sample surface was first wiped with 75% ethanol, and 40–50 mg of the material was cut into small pieces. Twenty-five milligrams of the silica gel-dried leaves or 40–50 mg of the roots were rubbed for two minutes at a frequency of 30 times/second in Mixer Mill MM400 (Retsch GmbH, Haan, Germany). The total genomic DNA was isolated from the crushed materials using the Plant Genomic DNA Kit (Tiangen Biotech (Beijing) Co, Ltd., Beijing, China) according to the manufacturer's instructions.

### Amplification, sequencing, and sequence analysis

Four markers, namely, ITS2, *psbA-trnH*, *matK*, and *rbcL*, were involved in the test (see details in supplementary data page 2, 10, 13, and 14). All of the steps were performed according to DNA barcoding standard operating procedures (DNA barcoding SOP), according to the China Plant BOL Group^24^ and Chen et al^25^,^27^.

## Author Contributions

J. S., H. Y. and S. C. conceived the study and participated in its design. T. X., X. L., Y. L., R. Y., L. N. and C. F. contributed samples and carried out the experiments. T. X. and X. M. analyzed the data. T. X., J. S., H. Y. and S. C. drafted the manuscript. All authors have read and approved the final manuscript.

## Supplementary Material

Supplementary Information Supplementary data

## Figures and Tables

**Table 1:** Sequence characteristics of the ITS2 barcodes of the specimens from the 10 *Rhodiola* species, authentic samples, and decoction pieces

	10 *Rhodiola* species	Authentic samples	Decoction pieces
Sequence length (bp)	216–223	217	216–219
G+C content range (mean) (%)	52.8–55.8 (54.2)	55.3–56.2 (55.5)	52.3–56.2 (55.4)
Variable sites	35	2	21
