# Supplementary material for: Survey of commercial Rhodiola products revealed species diversity and potential safety issues
Source: Sci Rep. 2015 Feb 9;5:8337. doi: 10.1038/srep08337 (PMC4321177; doi:10.1038/srep08337)
Supplement: Supplementary Information — Supplementary data [file srep08337-s1.pdf]

Survey of commercial *Rhodiola* products revealed species diversity and potential safety issues

Tianyi Xin[1*], Xiaojin Li[2*], Hui Yao[1], Yulin Lin[1], Xiaochong Ma[1], Ruiyang Cheng[3], Jingyuan Song[1, 4], Lianghong Ni[5], Congzhao Fan[2] & Shilin Chen[1, 3]

1. Institute of Medicinal Plant Development, Chinese Academy of Medical Sciences & Peking Union Medical College, Beijing 100193, P.R. China

2. Xinjiang Institute of Chinese Materia Medica and Ethnical Materia, Urumqi 830002, P.R. China

3. Institute of Chinese Materia Medica, China Academy of Chinese Medical Sciences, Beijing 100700, P.R. China

4. Chongqing Institute of Medicinal Plant Cultivation, Chongqing 408435, P.R. China

5. Shanghai University of Traditional Chinese Medicine, Shanghai 201203, P.R. China

[*] These authors contributed equally to this work.

**Corresponding author

Jingyuan Song, Professor

Address: No.151, Malianwa North Road, HaiDian District, Beijing 100193, P.R. China

Tel.: +86-10-57833199 Fax: +86-10-62811448

E-mail address: songjingyuan@hotmail.com

Shilin Chen, Professor

Address: No. 16 Nanxiaojie, Dongzhimennei Ave, Beijing, 100700, P.R. China

Tel.: +86-10-84017374

E-mail address: slchen@implad.ac.cn

Comparison among four DNA barcodes. We compared the divergence of ITS2, *psbA-trnH*, *rbcL*, and *matK* regions. For each of the ten species, one to five samples were selected for a supplementary test (Table S4). The results indicated the following:

(1) PCR amplification is highly successful for the ITS2 and *psbA-trnH* regions, whereas the *rbcL* and *matK* regions were not amplified in approximately one-third and one-fourth of the samples, respectively.

(2) All the PCR products of the ITS2, *psbA-trnH*, and *rbcL* regions could be bi-directional sequenced and assembled successfully, whereas only the reverse sequencing results could be obtained for the *matK* region because of the poly T region (with approximately 11 thymines).

(3) All the ITS2, *psbA-trnH*, *rbcL*, and *matK* sequences obtained in the experiment were aligned (Figure S1, S3-S5). Given that five species had no variability in the *rbcL* region (Figure S3) and the *matK* sequences of some samples could not be obtained in the experiment, these two markers (*rbcL* and *matK*) were deemed unsuitable for the identification of the ten *Rhodiola* species in this study. The *psbA-trnH* region could identify these species by BLAST1 analysis. Furthermore, ITS2 showed enough interspecies divergence to differentiate these species (main text page 5, line 25-28). The results demonstrated that the ITS2 barcode was able to accurately and swiftly distinguish the ten species.

(4) These results confirmed that the ITS2 and *psbA-trnH* regions were acceptably capable of species identification. These conclusions are consistent with those in our previous study [25]. Given that the use of one marker could solve the identification problem and minimize the cost in the experiment, we finally chose ITS2 as the DNA barcode in this study.

Table S1 List of specimens and authentic samples from the 10 *Rhodiola* species

Latin Name	Voucher No.	Haplotype	GenBank No.	DNA concentration (ng/μL)	OD260/280	Location
Rhodiola rosea	PS1750MT11	RR1	KJ796865	——	——	Qitai, Xinjiang
Rhodiola rosea	PS1750MT12	RR1		——	——	Jiminay, Xinjiang
Rhodiola rosea	PS1750MT13	RR1		——	——	Xinjiang
Rhodiola algida	PS1901MT01	RA1	KJ796854	371.6	1.97	Burqin, Xinjiang
Rhodiola fastigiata	PS1902MT01	RF1	KJ796857	32.1	1.59	Segrila Mountain, Tibet
Rhodiola fastigiata	PS1902MT02	RF2	KJ796858	46.7	1.47	Segrila Mountain, Tibet
Rhodiola fastigiata	PS1902MT03	RF2		20.8	1.68	Segrila Mountain, Tibet
Rhodiola fastigiata	PS1902MT04	RF2		53.2	1.46	Segrila Mountain, Tibet
Rhodiola gelida	PS1903MT01	RG1	KJ796859	19.7	1.16	Keriya, Xinjiang
Rhodiola gelida	PS1903MT02	RG2	KJ796860	——	——	Kuenes, Xinjiang
Rhodiola kirilowii	PS1904MT01	RK1	KJ796861	209.4	1.84	Urumchi, Xinjiang
Rhodiola kirilowii	PS1904MT02	RK1		——	——	Hejing, Xinjiang
Rhodiola kirilowii	PS1904MT03	RK1		——	——	Hejing, Xinjiang
Rhodiola kirilowii	PS1904MT04	RK1		——	——	Zhaosu, Xinjiang
Rhodiola kirilowii	PS1904MT05	RK1		——	——	Zhaosu, Xinjiang
Rhodiola kirilowii	PS1904MT06	RK1		——	——	Xinjiang
Rhodiola pamiro-alaica	PS1905MT01	RP1	KJ796862	——	——	Xinjiang
Rhodiola pamiro-alaica	PS1905MT02	RP1		——	——	Xinjiang
Rhodiola quadrifida	PS1906MT01	RQ1	KJ796863	92.3	1.35	Miquan, Xinjiang

Rhodiola quadrifida	PS1906MT02	RQ1		97.1	1.71	Fukang, Xinjiang
Rhodiola quadrifida	PS1906MT03	RQ1		88.5	1.38	Fukang, Xinjiang
Rhodiola quadrifida	PS1906MT04	RQ2	KJ796864	27.0	1.48	Burqin, Xinjiang
Rhodiola quadrifida	PS1906MT05	RQ1		296.4	1.38	Jimsar, Xinjiang
Rhodiola quadrifida	PS1906MT06	RQ1		357.0	2.02	Jimsar, Xinjiang
Rhodiola quadrifida	PS1906MT07	RQ3		——	——	Qitai, Xinjiang
Rhodiola quadrifida	PS1906MT08	RQ2		——	——	Jiminay, Xinjiang
Rhodiola sacra	PS1907MT01	RS1	KJ796866	46.7	1.18	Lhasa, Tibet
Rhodiola sacra	PS1907MT02	RS1		17.1	1.03	Lhasa, Tibet
Rhodiola serrata	PS1908MT01	RSE1	KJ796867	123.5	1.68	Beijing
Rhodiola crenulata	PS0576MT01	RC2	GQ434463	——	——	Wenchuan, Sichuan
Rhodiola crenulata	PS0576MT11	RC2		11.6	1.16	Segrila Mountain, Tibet
Rhodiola crenulata	PS0576MT12	RC2		42.6	1.08	Segrila Mountain, Tibet
Rhodiola crenulata	YC0141MT01	RC1	KJ796855	62.7	2.18	Naidong, Tibet
Rhodiola crenulata	YC0141MT02	RC2		228.1	1.57	Naidong, Tibet
Rhodiola crenulata	YC0141MT03	RC2		40.9	1.28	Naidong, Tibet
Rhodiola crenulata	YC0141MT04	RC2		56.9	1.68	Naidong, Tibet
Rhodiola crenulata	YC0141MT05	RC2		56.4	1.81	Naidong, Tibet
Rhodiola crenulata	YC0141MT06	RC1		9.6	1.54	Naidong, Tibet
Rhodiola crenulata	YC0141MT07	RC2		106.6	2.18	Naidong, Tibet
Rhodiola crenulata	YC0141MT08	RC2		84.5	1.87	Naidong, Tibet

Rhodiola crenulata	YC0141MT09	RC2		49.8	1.32	Naidong, Tibet
Rhodiola crenulata	YC0141MT10	RC1		130.8	1.68	Naidong, Tibet
Rhodiola crenulata	YC0141MT11	RC1		53.6	4.05	Sangri, Tibet
Rhodiola crenulata	YC0141MT12	RC3	KJ796856	27.4	1.85	Sangri, Tibet
Rhodiola crenulata	YC0141MT13	RC2		51.1	2.98	Sangri, Tibet
Rhodiola crenulata	YC0141MT14	RC2		116.9	1.65	Sangri, Tibet
Rhodiola crenulata	YC0141MT15	RC1		63.4	2.53	Sangri, Tibet
Rhodiola crenulata	YC0141MT16	RC1		16.8	1.53	Sangri, Tibet
Rhodiola crenulata	YC0141MT17	RC1		43.8	2.10	Sangri, Tibet
Rhodiola crenulata	YC0141MT18	RC1		72.1	5.06	Sangri, Tibet
Rhodiola crenulata	YC0141MT19	RC2		25.6	1.52	Sangri, Tibet
Rhodiola crenulata	YC0141MT20	RC3		24.3	1.17	Sangri, Tibet
Rhodiola crenulata	YC0141MT21	RC1		62.4	4.44	Linzhou, Tibet
Rhodiola crenulata	YC0141MT22	RC1		57.7	1.87	Linzhou, Tibet
Rhodiola crenulata	YC0141MT23	RC2		191.0	1.80	Linzhou, Tibet
Rhodiola crenulata	YC0141MT24	RC1		134.1	2.28	Linzhou, Tibet
Rhodiola crenulata	YC0141MT25	RC1		2.6	2.26	Linzhou, Tibet
Rhodiola crenulata	YC0141MT26	RC1		15.2	2.40	Linzhou, Tibet
Rhodiola crenulata	YC0141MT27	RC1		33.1	1.69	Linzhou, Tibet
Rhodiola crenulata	YC0141MT28	RC1		175.6	2.23	Linzhou, Tibet
Rhodiola crenulata	YC0141MT29	RC1		69.3	2.73	Linzhou, Tibet

Rhodiola crenulata	YC0141MT30	RC1	33.3	2.26	Linzhou, Tibet
Rhodiola crenulata	YC0141MT31	RC1	40.1	1.63	Linzhou, Tibet
Rhodiola crenulata	YC0141MT32	RC1	43.7	3.80	Linzhou, Tibet
Rhodiola crenulata	YC0141MT33	RC3	53.1	1.78	Linzhou, Tibet
Rhodiola crenulata	YC0141MT34	RC1	45.7	1.52	Qushui, Tibet
Rhodiola crenulata	YC0141MT35	RC1	149.7	2.50	Qushui, Tibet
Rhodiola crenulata	YC0141MT36	RC2	57.8	1.49	Qushui, Tibet
Rhodiola crenulata	YC0141MT37	RC1	64.0	1.52	Qushui, Tibet
Rhodiola crenulata	YC0141MT38	RC2	90.2	1.56	Qushui, Tibet
Rhodiola crenulata	YC0141MT39	RC1	6.1	2.02	Qushui, Tibet
Rhodiola crenulata	YC0141MT40	RC1	102.2	2.05	Qushui, Tibet
Rhodiola crenulata	YC0141MT41	RC1	161.6	2.05	Qushui, Tibet
Rhodiola crenulata	YC0141MT42	RC2	11.6	0.90	Qushui, Tibet
Rhodiola crenulata	YC0141MT43	RC2	866.4	0.89	Qushui, Tibet
Rhodiola crenulata	YC0141MT44	RC1	6.9	1.23	Qushui, Tibet
Rhodiola crenulata	YC0141MT45	RC2	207.5	1.26	Chengdu, Sichuan
Rhodiola crenulata	YC0141MT46	RC2	5.3	1.07	Chengdu, Sichuan
Rhodiola crenulata	YC0141MT47	RC1	83.3	1.63	Chengdu, Sichuan
Rhodiola crenulata	YC0141MT48	RC2	50.3	1.86	Chengdu, Sichuan
Rhodiola crenulata	YC0141MT49	RC2	3.2	0.99	Chengdu, Sichuan
Rhodiola crenulata	YC0141MT50	RC2	4.9	1.04	Chengdu, Sichuan

Table S2 Commercial decoction piece samples sold as Rhodiolae Crenulatae Radix et Rhizoma from different provinces in China

Sample No.	Best hit	Purchased Place	Sample No.	Best hit	Purchased Place
HYP01	*Rhodiola crenulata*	Beijing	HYP21	*Rhodiola rosea*	Wuhan, Hubei
HYP02	*Rhodiola serrata*	Beijing	HYP22	*Rhodiola crenulata*	Wuhan, Hubei
HYP03	*Rhodiola crenulata*	Beijing	HYP23	*Rhodiola crenulata*	Changsha, Hunan
HYP04	*Rhodiola serrata*	Beijing	HYP24	*Rhodiola gelida*	Changsha, Hunan
HYP05	*Rhodiola quadrifida*	Xushui, Hebei	HYP25	*Rhodiola gelida*	Changsha, Hunan
HYP06	*Rhodiola fastigiata*	Baoding, Hebei	HYP26	*Rhodiola gelida*	Guangzhou, Guangdong
HYP07	*Rhodiola serrata*	Baoding, Hebei	HYP27	*Rhodiola serrata*	Guangzhou, Guangdong
HYP08	*Rhodiola serrata*	Chengdu, Sichuan	HYP28	*Rhodiola crenulata*	Guangzhou, Guangdong
HYP09	*Rhodiola crenulata*	Chengdu, Sichuan	HYP29	*Rhodiola serrata*	Guangzhou, Guangdong
HYP10	*Rhodiola crenulata*	Chengdu, Sichuan	HYP30	*Rhodiola gelida*	Sanya, Hainan
HYP11	*Rhodiola crenulata*	Chengdu, Sichuan	HYP31	*Rhodiola serrata*	Sanya, Hainan
HYP12	*Rhodiola crenulata*	Chengdu, Sichuan	HYP32	*Rhodiola crenulata*	Sanya, Hainan
HYP13	*Rhodiola crenulata*	Tianjin	HYP33	*Rhodiola crenulata*	Sanya, Hainan
HYP14	*Rhodiola crenulata*	Xichang, Sichuan	HYP34	*Rhodiola crenulata*	Kunming, Yunnan
HYP15	*Rhodiola crenulata*	Urumchi, Xinjiang	HYP35	*Rhodiola serrata*	Kunming, Yunnan
HYP16	*Rhodiola rosea*	Urumchi, Xinjiang	HYP36	*Rhodiola serrata*	Kunming, Yunnan
HYP17	*Rhodiola serrata*	Urumchi, Xinjiang	HYP37	*Rhodiola crenulata*	Kunming, Yunnan
HYP18	*Rhodiola crenulata*	Wuhan, Hubei	HYP38	*Rhodiola gelida*	Hefei, Anhui
HYP19	*Rhodiola crenulata*	Beijing	HYP39	*Rhodiola serrata*	Hefei, Anhui
HYP20	*Rhodiola crenulata*	Wuhan, Hubei	HYP40	*Rhodiola rosea*	Hefei, Anhui

HYP41	*Rhodiola serrata*	Guiyang, Guizhou	HYP62	*Rhodiola crenulata*	Hangzhou, Zhejiang
HYP42	*Rhodiola serrata*	Guiyang, Guizhou	HYP63	*Rhodiola rosea*	Shanghai
HYP43	*Rhodiola crenulata*	Guiyang, Guizhou	HYP64	*Rhodiola serrata*	Shanghai
HYP44	*Rhodiola serrata*	Taiyuan, Shanxi	HYP65	—	Shanghai
HYP45	*Rhodiola serrata*	Taiyuan, Shanxi	HYP66	—	Shanghai
HYP46	*Rhodiola serrata*	Taiyuan, Shanxi	HYP67	—	Changchun, Jilin
HYP47	*Rhodiola serrata*	Xiaoyi, Shaanxi	HYP68	*Rhodiola serrata*	Changchun, Jilin
HYP48	*Rhodiola serrata*	Xiaoyi, Shaanxi	HYP69	*Rhodiola rosea*	Changchun, Jilin
HYP49	*Rhodiola serrata*	Xiaoyi, Shaanxi	HYP70	*Rhodiola serrata*	Yinchuan, Ningxia
HYP50	*Rhodiola rosea*	Huaian, Jiangsu	HYP71	*Rhodiola serrata*	Yinchuan, Ningxia
HYP51	*Rhodiola quadrifida*	Huaian, Jiangsu	HYP72	*Rhodiola gelida*	Yinchuan, Ningxia
HYP52	*Rhodiola serrata*	Huaian, Jiangsu	HYP73	*Rhodiola serrata*	Yinchuan, Ningxia
HYP53	*Rhodiola serrata*	Xiamen, Fujian	HYP74	*Rhodiola crenulata*	Luoyang, Henan
HYP54	*Rhodiola crenulata*	Xiamen, Fujian	HYP75	*Rhodiola rosea*	Luoyang, Henan
HYP55	*Rhodiola crenulata*	Xiamen, Fujian	HYP76	*Rhodiola serrata*	Luoyang, Henan
HYP56	*Rhodiola crenulata*	Chongqing	HYP77	*Rhodiola crenulata*	Xining, Qinghai
HYP57	*Rhodiola crenulata*	Chongqing	HYP78	—	Xining, Qinghai
HYP58	*Rhodiola crenulata*	Chongqing	HYP79	*Rhodiola serrata*	Xining, Qinghai
HYP59	—	Hangzhou, Zhejiang	HYP80	*Rhodiola crenulata*	Guilin, Guangxi
HYP60	—	Hangzhou, Zhejiang	HYP81	*Rhodiola gelida*	Guilin, Guangxi
HYP61	*Rhodiola serrata*	Hangzhou, Zhejiang	HYP82	*Rhodiola crenulata*	Guilin, Guangxi

HYP83	*Rhodiola crenulata*	Guilin, Guangxi	HYP92	—	Nanchang, Jiangxi
HYP84	*Rhodiola serrata*	Qingdao, Shandong	HYP93	*Rhodiola crenulata*	Shenyang, Liaoning
HYP85	*Rhodiola rosea*	Qingdao, Shandong	HYP94	*Rhodiola crenulata*	Shenyang, Liaoning
HYP86	*Rhodiola serrata*	Qingdao, Shandong	HYP95	*Rhodiola crenulata*	Shenyang, Liaoning
HYP87	—	Taipei, Taiwan	HYP96	—	Harbin, Heilongjiang
HYP88	—	Taipei, Taiwan	HYP97	*Rhodiola rosea*	Urumchi, Xinjiang
HYP89	*Rhodiola serrata*	Taipei, Taiwan	HYP98	*Rhodiola serrata*	Kunming, Yunnan
HYP90	*Rhodiola crenulata*	Shangrao, Jiangxi	HYP99	*Rhodiola crenulata*	Chengdu, Sichuan
HYP91	*Rhodiola serrata*	Nanchang, Jiangxi	HDP01	*Rhodiola serrata*	Xichang, Sichuan

Symbol "—" indicated the sample did not get ideal DNA barcode sequence

Table S3 List of *Rhodiola* species that contain cyanogenic glycosides

	**Latin name**
***Rhodiola* species contain cyanogenic clycosides**	*Rhodiola rosea* L.; *R. gelida* Schrenk; *R. quadrifida* (Pall.) Fisch. et Mey.; *R. concinna* (Praeg.) S. H. Fu; *R. kirilowii* (Regel) Maxim.; *R. junggarica* C. Y. Yang et N. R. Cui; *R. linearifolia* A. Bor.; *R. litwinowii* A. Bor.; *R. pamiro-alaica* A. Bor.; *R. heterodonta* (Hook. f. et Thoms.) A. Bor.; *R. bupleuroides* (Wall. ex Hook. f. et Thoms.) S. H. Fu;

Table S4 Sequence recovery of the four barcodes in the 10 *Rhodiola* species

Voucher No.	Latin name	ITS2	*psbA-trnH*	*matK*	*rbcL*
PS1901MT01	*Rhodiola algida*	√	√	√	√
PS0576MT11	*Rhodiola crenulata*	√	√	—	—
YC0141MT08	*Rhodiola crenulata*	√	√	√	√
YC0141MT09	*Rhodiola crenulata*	√	√	—	√
PS1902MT01	*Rhodiola fastigiata*	√	√	—	—
PS1902MT02	*Rhodiola fastigiata*	√	√	—	—
PS1902MT03	*Rhodiola fastigiata*	√	√	—	—
PS1902MT04	*Rhodiola fastigiata*	√	√	—	—
PS1903MT02	*Rhodiola gelida*	√	√	√	√
PS1904MT02	*Rhodiola kirilowii*	√	√	√	√
PS1904MT03	*Rhodiola kirilowii*	√	√	√	√
PS1904MT04	*Rhodiola kirilowii*	√	√	√	√
PS1904MT05	*Rhodiola kirilowii*	√	√	√	√
PS1904MT06	*Rhodiola kirilowii*	√	√	√	√
PS1905MT01	*Rhodiola pamiro-alaica*	√	√	√	√
PS1905MT02	*Rhodiola pamiro-alaica*	√	√	—	√
PS1906MT01	*Rhodiola quadrifida*	√	√	√	√
PS1906MT04	*Rhodiola quadrifida*	√	√	√	√
PS1906MT07	*Rhodiola quadrifida*	√	√	√	√
PS1750MT11	*Rhodiola rosea*	√	√	√	√
PS1750MT12	*Rhodiola rosea*	√	√	√	√
PS1750MT13	*Rhodiola rosea*	√	√	√	√
PS1907MT01	*Rhodiola sacra*	√	√	√	√
PS1907MT02	*Rhodiola sacra*	√	√	√	√
PS1908MT01	*Rhodiola serrata*	√	√	—	—

Symbol “ √ ” refers to the samples with the relevant barcode sequence

Symbol “—” refers to the samples which do not have the relevant barcode sequence

```
                                                 11111111 1111111111 22222
                                     12333568 9900122335 5556677899 00112
                                   2628248174 7919468490 5690113746 09195
Rhodiola crenulata     PS0576MT01  GCGGTGTCCC GTACCAGCCT GCCGCCCTCC CCTCT
Rhodiola algida        PS1901MT01  .T.C...... ........T. .TT....... .TC..
Rhodiola fastigiata    PS1902MT01  AT.....A.. T...TGAATG .T........ GGC.C
Rhodiola fastigiata    PS1902MT02  AT.....A.. T...TGAATG .T........ G.C..
Rhodiola gelida        PS1903MT02  ........T. ........T. .......... .....
Rhodiola gelida        PS1903MT01  .......... ........T. ........A. .....
Rhodiola kirilowii     PS1904MT02  .T........ ...T.G..TG AT..T..... ..C..
Rhodiola pamiroalaica  PS1905MT01  AT.....A.. T...T.A.TG .T........ G.C..
Rhodiola quadrifida    PS1906MT07  .T........ ...Y.G..TG .T..T..... ..CT.
Rhodiola quadrifida    PS1906MT08  .TA....... .....G..TG .T..T....T ..C..
Rhodiola quadrifida    PS1906MT01  .T........ .....G..TG .T..T..... ..CT.
Rhodiola rosea         PS1750MT01  AT.....A.. T...T.A.TG .T........ G.C..
Rhodiola sacra         PS1907MT01  .T..CAC..G ..TT....T. .T.CTTGA.. ..C..
Rhodiola serrata       PS1908MT01  .......... .C..TGA.TG .......... .....
```

Figure S1 Variable sites in the ITS2 sequence of the 10 *Rhodiola* species

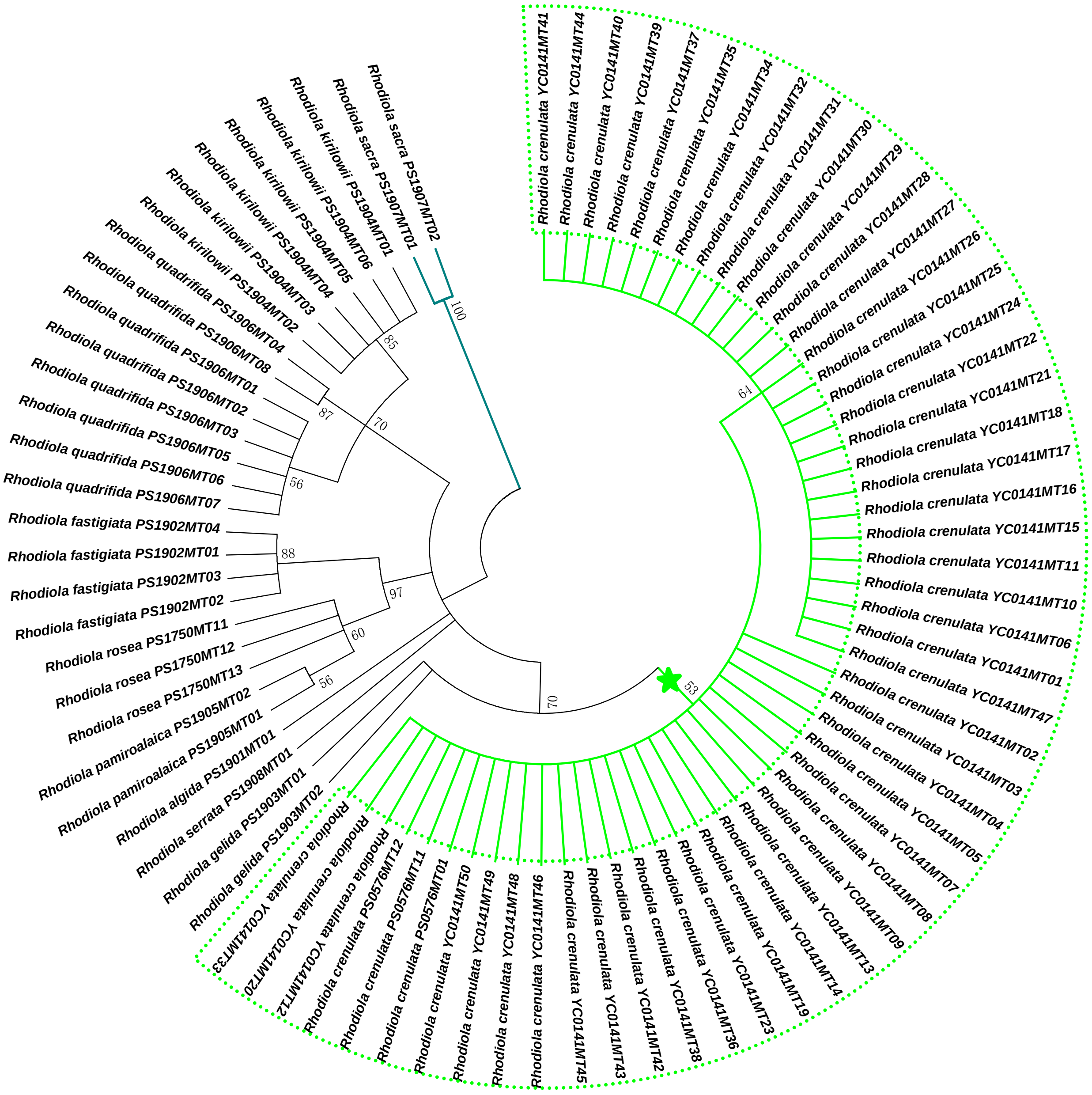

Figure S2　NJ tree of *Rhodiola crenulata* and its closely related species constructed using the ITS2 sequences. The bootstrap scores (1 000 replicates) are shown (≥50%) for each branch.　　Symbol ★ showed the clade of *R. crenulata*

		4556
		47595
		63561
Rhodiola kirilowii	PS1904MT02	CTCGA
Rhodiola kirilowii	PS1904MT03	.....
Rhodiola kirilowii	PS1904MT04	.....
Rhodiola kirilowii	PS1904MT05	.....
Rhodiola kirilowii	PS1904MT06	.....
Rhodiola quadrifida	PS1906MT01	.....
Rhodiola quadrifida	PS1906MT04	.....
Rhodiola quadrifida	PS1906MT07	.....
Rhodiola gelida	PS1903MT02	.....
Rhodiola crenulata	YC0141MT08	.....
Rhodiola crenulata	YC0141MT09	.....
Rhodiola pamiro-alaica	PS1905MT01	.....
Rhodiola pamiro-alaica	PS1905MT02	.....
Rhodiola rosea	PS1750MT11	.C...
Rhodiola rosea	PS1750MT12	.....
Rhodiola rosea	PS1750MT13	.C...
Rhodiola algida	PS1901MT01	..T..
Rhodiola sacra	PS1907MT01	A.TTC
Rhodiola sacra	PS1907MT02	A.TTC

Figure S3 Variable sites of all the *rbcL* sequences in the test.

		1122 2333344455 5555666667 777
		1779991937 9336715600 2599366781 134
		2061491127 6561639705 7823657653 705
Rhodiola quadrifida	PS1906MT01	TTACGCGAGC GCCAGGGGGG AACTGTAAGC TCG
Rhodiola quadrifida	PS1906MT04	.......G.. ..T....... ....
Rhodiola quadrifida	PS1906MT07	....
Rhodiola gelida	PS1903MT02	C.GA...... T......... ...C...T.. ...
Rhodiola pamiro-alaica	PS1905MT01	..GA.....T T......A.. .G....GT.. ...
Rhodiola algida	PS1901MT01	.AGA.AC.T. TA..A..... ....T.. ...
Rhodiola crenulata	YC0141MT08	..GA.....T T......... ....A..... C..
Rhodiola rosea	PS1750MT11	..G....... T.......A. C.A....T.. ...
Rhodiola rosea	PS1750MT12	..G....... T......... ..A....T.. ...
Rhodiola rosea	PS1750MT13	..G.A..... T.......A. C.A....T.. ...
Rhodiola kirilowii	PS1904MT03	..G....... T....T.... ....T ...
Rhodiola kirilowii	PS1904MT02	..G....... T....T.... ....T ...
Rhodiola kirilowii	PS1904MT04	..G....... T.....A... ....A. .T.
Rhodiola kirilowii	PS1904MT05	..G....... T......... ..A....T.. ...
Rhodiola kirilowii	PS1904MT06	..G....... T......... ....TA. .T.
Rhodiola sacra	PS1907MT01	.AG...C... T..GA..... .....G.T.. ..A
Rhodiola sacra	PS1907MT02	.AG...C... T..GA....T .....G.T.. ..A

Figure S4 Variable sites of all the *matK* sequences in the test.

		11 1111111111 1122222222 2222222222 2222223333 33
		467789900 0001224688 9900133444 5567788888 8889990000 13
		5094590536 7892256513 4623608289 1983502356 7890241378 50
Rhodiola algida	PS1901MT01	CCCCGACGCG AAAGCGAGAA CATGTAAGCC GTTT------ ----GT---- -G
Rhodiola crenulata	PS0576MT11	...T..A..T TTC..T..C. .CCT.....T ....C----- ----AAGA-- -.
Rhodiola crenulata	YC0141MT08	......A..T TTC..T..C. TCCT.....T ....CTTAAT ATAT--GATC G.
Rhodiola crenulata	YC0141MT09	......A... .....T..C. .CCTC....T ....CAATTC TATAAAGATC G.
Rhodiola fastigiata	PS1902MT01	T...TGA... .....TT.C. ..CT..T..T ....------ ----AAGA-- -.
Rhodiola fastigiata	PS1902MT02	......A..T TTC..T..C. ..CT.....T ....C----- ----AATATA G.
Rhodiola fastigiata	PS1902MT03	......A..T TTC..T..C. ..CT.....T ....C----- ----AATATA G.
Rhodiola fastigiata	PS1902MT04	......A..T TTC..T..C. ..CT.....T ....A----- ----AATATA G.
Rhodiola gelida	PS1903MT02	......A..T TTC..T.TC. ----.....T ..A.ATTAAT ATATAATATC G.
Rhodiola kirilowii	PS1904MT02	......AA.T TTC..T..C. ..AT.....T A..AA----- ----AAGATC G.
Rhodiola kirilowii	PS1904MT03	......AA.T TTC..T..C. ..AT.....T A..AA----- ----AAGATC G.
Rhodiola kirilowii	PS1904MT04	......A..T TTC..T..C. ..CT.....G A...A----- ----AAGATC T.
Rhodiola kirilowii	PS1904MT05	.T....A..T TTC..T..C. ..CT.....T A.A.A----- ----AA-ATC G.
Rhodiola kirilowii	PS1904MT06	...T..A... .....T..CC ..AT.G..TG A...C----- ----AATATA G.
Rhodiola pamiro-alaica	PS1905MT01	...T..A..T TTC..T..C. .CCT.....T ....A----- ----AAGATC G.
Rhodiola pamiro-alaica	PS1905MT02	......A..T TTC..T.TC. ----.....T ..A.ATTAAT AT----TATC G.
Rhodiola quadrifida	PS1906MT01	......A... .....T..CC ..CT.....T ....A----- ----A.TTTC G.
Rhodiola quadrifida	PS1906MT04	......A..T TTC..T..CC ..CT.....T ....A---TT TATAAA--TC G.
Rhodiola quadrifida	PS1906MT07	......A... .....T..CC ..CT.....T ....A----- ----A.TTTC G.
Rhodiola rosea	PS1750MT11	..T...A.TT TTC..T..C. ..CT.....T A.A.A---TT TATAAAGATC G.
Rhodiola rosea	PS1750MT12	..T...A.T. ...A.T..C. ..CT.....T A...A---TT TATAAAGATC G.
Rhodiola rosea	PS1750MT13	..T...A.TT TTC..T..C. ..CT.....T A...A---TT TATAAAGATC G.
Rhodiola sacra	PS1907MT01	......A... ....TT..CC ..AT...A.. .G..------ ----..---- -A
Rhodiola sacra	PS1907MT02	......A... ....TT..C. ..AT...A.. ....------ ----..---- -A
Rhodiola serrata	PS1908MT01	T....GA... .....TT.C. ..CT..T..T ....------ ----AAGACA G.

Figure S5 Variable sites of all the *psbA-trnH* sequences in the test.
